# Supplementary material for: Evaluation of the first edition of the ‘Spirituality in medicine’ programme implemented for medical students at the Nicolaus Copernicus University, Collegium Medicum in Bydgoszcz, Poland. Pre- and post-test research
Source: BMC Med Educ. 2026 May 28;26:1210. doi: 10.1186/s12909-026-09439-6 (PMC13404866; doi:10.1186/s12909-026-09439-6)
Supplement: Supplementary file 1 — Supplementary Material 1. [file 12909_2026_9439_MOESM1_ESM.docx]

**Title:** **Evaluation of the First Edition of the ‘Spirituality in Medicine’ Programme Implemented for Medical Students at the Nicolaus Copernicus University, Collegium Medicum in Bydgoszcz, Poland. Pre- and post-test research**

**Authors:**

1. Małgorzata Fopka-Kowalczyk, Department of Philosophy and Social Science, Nicolaus Copernicus University, Toruń, Poland; e-mail address: [mfopka-kowalczyk@umk.pl](mailto:mfopka-kowalczyk@umk.pl). **Corresponding author.**
2. **Małgorzata Krajnik,** Department of Palliative Care, Collegium Medicum in Bydgoszcz, Nicolaus Copernicus University in Toruń, Poland; e-mail address: [malgorzata.krajnik@cm.umk.pl](mailto:malgorzata.krajnik@cm.umk.pl)

**Abstract:**

**Background:** This article presents an evaluation of the first obligatory programme on ‘Spirituality in Medicine’ for medical students in Poland, implemented at the Collegium Medicum in Bydgoszcz of the Nicolaus Copernicus University in Toruń. **Methods:** The main research question was: To what extent did participation in the ‘Spirituality in Medicine’ programme influence the level of competence in spiritual care among the students taking part in the project? To assess spirituality competencies, the study employed the Spiritual Supporter Scale, and evaluation was performed before, during, and after completing the educational programme on spiritual care. **Participants:** A pre–post single-group intervention design without a control group was employed. **121 participants comprised the m**edical students of the Collegium Medicum in Bydgoszcz at the Nicolaus Copernicus University in Toruń, Poland who participated in the first edition of the ‘Spirituality in Medicine’ module implemented from the second to the fifth year of general education (2018/2019 – 2021/2022) (250). **Results: T**he program was associated with modest changes in select subdomains of the SpSup scale, while other domains showed insignificant changes or declines. The results of the Friedman ANOVA test shows the statistically significant changes in the two subscales: spirituality in relation to one’s own suffering and that of others (χ2=7.47; p<0.05), and the recognition of spiritual suffering (χ2=8.89; p<0.05), however effect sizes in both of subdomains were small. In the third subscale, prayer as support, the level of competence was higher before the training than after its completion (χ2 = 6.09, p<0.05). In the remaining measurements, an increase in results was observed in the second measurement, but these differences are not statistically significant. The results of this statistics showed that the only statistically significant difference occurred in the factor of community treated as a support system in the pair of measurements before and during education (p<0.05), however the size effect was small. The other measurements, related to beliefs about spirituality and community as support in the during-after measurement, proved to be statistically insignificant. **Conclusion:** The introduction of the ‘Spirituality in Medicine’ programme in the general education curriculum for medical students has an impact on the level of competencies in the areas of spirituality in relation to one’s own suffering and that of others and in the field of recognition of spiritual suffering in a pre-post-test study and in the contrast method in the field of community treated as a support system in the pair of measurements before and during education. Results show, that the programme has to be corrected and evaluated, particularly in terms of improving the quality of the content offered and self-reflection.

**Keywords:** First training programme in Poland, spirituality curriculum, Spirituality in Medicine, medical students, evaluation.

**Background**

Contemporary medical care increasingly addresses issues concerning a holistic approach to patients and their experiences with illness. Not only is the disease important per se, but also the emotional aspect of a person’s life and the social dimension. Equally important is the spiritual dimension of the sick person, the recognition of which enables a deeper understanding of the patient and yields numerous benefits to both parties in the relationship. A holistic approach to patient care also responds to the needs of patients, for whom the doctor or other healthcare professional, besides knowledge, should possess qualities such as attentiveness, patience, a lack of indifference to the patient’s suffering, and the ability to provide compassionate care. Patients also expect to be listened to and for their experiences and spiritual needs to be acknowledged and addressed [1]. Moreover, satisfactory spiritual care for the patient has a favourable impact on survival [2], coping with disease [3], patient satisfaction with treatment and care [4], compliance with treatment [5], greater well-being and quality of life [6–10], as well as reduced anxiety and depression [11–15]. Patients are also better able to cope with their disease and maintain a more positive attitude despite their sickness [3, 15]. All these relationships confirm that spirituality is an indispensable part of human life and patient care [16-18].

There are several concepts of spirituality in medicine. Two of them formed the basis of the pre-research and the counter-tool. One is described by the European Association for Palliative Care (EAPC) Task Force in 2011 (revised in 2020), thus: ‘spirituality is the dynamic dimension of human life that relates to the way persons (individual and community) experience, express and/or seek meaning, purpose and transcendence, and the way they connect to the moment, to self, to others, to nature, to the significant and/or the sacred’ (revised definition from 2011) [19]. The second concept was developed by the Polish Association for Spiritual Care in Medicine (PASCiM), which defines spirituality as a dimension of human life that concerns transcendence and other existentially important values [20, 21]. Based on the EAPC approach to spirituality, PASCiM similarly recognises three dimensions of spiritual experience, which include the religiousness of a person, especially their relationship with God, personal beliefs, and religious practices, as well as community interaction; existential quest, particularly regarding the meaning of life, suffering, and death, issues of dignity, personhood, a sense of individual freedom and responsibility, hope and despair, reconciliation and forgiveness, love and joy; and the values by which a person lives, especially concerning oneself and others, work, nature, art and culture, ethical and moral choices, and life itself [19 - 21].

Based on these theoretical assumptions and in collaboration with specialists in spirituality outside Poland, a spirituality teaching programme was created for medical students. The first compulsory spiritual care programme offered in a medical school in Poland was launched in 2017 at the Collegium Medicum in Bydgoszcz, Nicolaus Copernicus University in Toruń [22]. The Department of Palliative Care introduced it during the academic year 2018/2019 . It envisioned work with medical students throughout the subsequent years of their studies (i.e. from the 2^nd^ to the 5^th^ year, totalling 48 teaching hours) (Table 1).

This article aim was to evaluate how participation in the first edition of the programme influences students’ knowledge and attitudes towards spiritual suffering and spiritual care for their patients. We hope that the current analysis of this programme’s effects on students’ competence and sensitivity will enable us to answer the research objective and questions set out below.

Table 1. Outline of the Obligatory Programme of Education in Spirituality for Medical Students at the Collegium Medicum in Bydgoszcz, Nicolaus Copernicus University in Toruń

| **Programme Implemented in 2018/2019** | | | | |
| --- | --- | --- | --- | --- |
| Form | 2^nd^ Year  (n = 189) | 3^rd^ Year  (n = 177) | 4^th^ Year  (n = 177) | 5^th^ Year(n = 175) |
|  | Introduction to spirituality in medicine, Basic spiritual care provided by doctors, Specialist spiritual care, Total pain, spiritual pain, and suffering, Compassion in clinical practice, Communication and personal pathway to becoming a doctor. | Mindful presence or spirituality in clinical practice, Diagnosing spiritual needs, Nonviolent communication. | Helping families say goodbye to loved ones dying alone in hospital due to COVID-19. Spiritual care for patients with COVID-19. Hope from a psychologist’s perspective. Cooperation with the chaplain, Dignity therapy, How doctors can support patients. | Developing a hospital programme on spiritual care, Helping find meaning, Communication about spirituality. |
| Lectures | 4 | 4 | - | - |
| Seminars | 4 | 4 | 8 | 4 |
| Workshops | 4 | 4 | 4 | 8 |

**Methods**

***The Aim of the Study***

The research project aimed to verify whether and in what extend the ‘Spirituality in Medicine’ programme introduced in the medical faculty had caused a change in the level of spiritual competence among the student participants (Table 1). An additional objective, due to the four-year duration of education in this field, which enabled an extended programme, was to assess the level of spiritual care skills after each block of education, starting with beliefs about spirituality, through sensitivity to spiritual suffering, and ending with the ability to provide support and spiritual care.

**The main research question is:**

To what extent did participation in the ‘Spirituality in Medicine’ programme influence the level of competence in spiritual care, as measured on the SuppScale, among the students taking part in the programme?

Based on the main research question the study considered **the following specific questions**:

1. What is the level of spiritual competence among medical students in the first edition of the ‘Spirituality in Medicine’ course?

2. What are the differences between the average scores obtained by students on the SpSup Scale before and after completing the programme?

3. What are the differences in the level of competence measured using the SpSup Scale among students before the introduction of the programme, during its implementation, and after its completion?

***Measures***

To achieve the research objective, as no suitable research tool was available in Polish, the Spiritual Supporter Scale (SpSup Scale) was developed and standardised by Małgorzata Fopka-Kowalczyk and Małgorzata Krajnik [23]. The SpSup Scale consists of 31 items assigned to five subscales that enable the examination of spiritual competence: 1) beliefs regarding spirituality, 2) sensitivity and recognition of spiritual suffering, 3) prayer as a support system, 4) community as a system of spiritual support, and 5) spirituality as a phenomenon. For the whole scale, the value is α = 0.88, while for individual subscales it is 1 = 0.65; 2 = 0.85; 3 = 0.84; 4 = 0.73; 5 = 0.73 (23). The survey included a form with questions about the respondents’ sex, age, and year of study.

***Participants***

The present study employed a pre–post single-group intervention design without a control group. The participants in the study were medical students from the Collegium Medicum in Bydgoszcz, Nicolaus Copernicus University in Toruń, who had taken part in the first edition of the ‘Spirituality in Medicine’ module, implemented from the second to the fifth year of general education (2018/2019 – 2021/2022). The research was conducted online in the following stages, in line with the content introduced during the course:

1) The whole SpSup Scale test – before starting the programme – at the beginning of the second year of education;

2) The SpSup Scale subscale examining beliefs about spirituality – at the beginning of the third year;

3) The SpSup Scale subscale examining the ability to recognise spiritual suffering – after the third year ;

4) The SpSup Scale subscale assessing students’ competences in spiritual support and care – after the fourth year;

5) The entire SpSup scale after completion of education in the fifth year of study.

**Survey administration.** The survey was conducted from October 2018 to March 2022. The questionnaire was designed using Nicolaus Copernicus University platform - *ankiety.umk.pl*. All students in each year were invited to complete the questionnaire voluntary and anonymous and registration form before the start of spirituality classes. However, as the selected data analysis methods require the same group of people whose results can be analysed, the number of students who completed the scale for all the indicated years was checked. Individuals in the study were identified by entering a PIN, which students entered into the completed questionnaire each time they logged in. An additional security measure was an individual TOKEN assigned to each respondent, enabling identification of whether that person had participated in previous measurements. Notably, the selected technique did not allow the identification of the respondents. Ultimately, 121 students who completed the test at each stage of their education (i.e. 69% of all 175 fifth-year students) were included in the statistical analyses (Table 1). The smaller number of questionnaires used for statistical analysis stems from the principles governing the use of pre- and post-tests, which involve analysing only the results of respondents who completed the questionnaires in each of the periods under study. Based on Ferguson and Takane analysis, this type of study is a single-factor study, where there is only one group and each person is studied several times (24). During the initial analysis of the data, the authors did not take into account questionnaires completed in only one period (e.g. only in the pre-test or only in the post-test), or those completed incorrectly or left blank, which would have precluded analysis. It is worth noting that the voluntary and anonymous nature of the study gave participants the option to withdraw from the test at any stage.

***Ethics approval***

The study was approved by the Bioethics Committee at Nicolaus Copernicus University in Toruń, Collegium Medicum in Bydgoszcz in 2018 (KB 736/2018) and the Ethics Committee of the Faculty of Philosophy and Social Sciences at Nicolaus Copernicus University in Toruń in 2020 (No. 1/2020). According to the Polish law and Good Clinical Practice, the project was not classified as a medi­cal experiment. The study was conducted in accordance with the principles of the Declaration of Helsinki. Ethics committees have approved the research without requiring the participants’ consent. The research data are presented per the APA 7^th^ edition standards [25].

***Informed consent to participate in the study***

Students were informed taking part in study was voluntary and anonymous and all collected answers would be used exclusively for scientific purposes. Their decision to complete the questionnaire was equivalent to their consent to participate in the research. The voluntary and anonymous nature of the study gave participants the option to withdraw from the test at any stage without any consequences. To make research more anonymous, we used also PIN technics, which is described above. Notably, the selected technique did not allow the identification of the respondents.

***Methods of Analysis***

The following statistical analyses were performed using JASP v0.18.3 2024 [26]:

- Wilcoxon test for dependent groups – to determine whether the results of the first measurement of spiritual competence levels using the SpSup Scale differ from the values obtained in the second study, and whether these differences are statistically significant. This test was chosen because the results of the Shapiro-Wilk Test indicate a distribution that deviates significantly from the normal distribution (Table 2).

- ANOVA test for more than three Friedman measurements – to examine the impact of the introduced programme on the level of spiritual competence in factors expressed in the individual subscales of the SpSup Scale: beliefs about spirituality, spirituality as a phenomenon, recognition of spiritual suffering, community as a support system, and prayer as a support system, in three measurements: before, during and after the programme, and to estimate the significance of the differences between the measurements obtained;

- Conover repeated measures test – to estimate statistically significant differences between fixed pairs of measurements when the Friedman ANOVA test values are statistically significant.

- Contrast method – to estimate statistically significant differences between fixed pairs of measurements when the Friedman ANOVA test values are statistically insignificant (27).

- Sex – enabling the assessment of differences in competence between women and men.

***Results***

*Sample Description*

A hundred and twenty-one students who began their medical education in the 2017/2018 academic year and completed the test at each stage of their education (i.e. 69% of 175 fifth-year students) were included in the statistical analyses (Table 1).

Table 1. Sample Description

|  | **Sex** | | **Age at the Beginning and End of the Programme** | |
| --- | --- | --- | --- | --- |
|  | F | M | II Year | V Year |
| **N** | 80 | 41 | 121 | 121 |
| **%** | 66.12% | 33.88% | - | - |
| **M** | - | - | 21.11 | 24 |
| **SD** | - | - | 1.6 | 1.63 |
| **Max** | - | - | 28 | 31 |
| **Min** | - | - | 19 | 22 |

The respondents comprised 80 women (66.12%) and 41 men (33.88%). The age of the respondents was presented based on the data obtained before and after the spirituality programme. The respondents’ average age before the start of the educational programme was M = 21.11, SD = 1.6, while after its completion, it was M = 24, SD = 1.63. The respondents’ age ranged from 19 to 28 before the start of the programme and from 22 to 31 after its completion.

Table 2. Normality Test – Shapiro-Wilk test

|  | |  | |  | | | **W** | | **p** | |
| --- | --- | --- | --- | --- | --- | --- | --- | --- | --- | --- |
| OR PRE |  | - |  | OR POST | |  | 0.988 |  | 0.334 |  |
| Beliefs PRE |  | - |  | Beliefs POST | |  | 0.977 |  | 0.039 |  |
| Recogn PRE |  | - |  | Recogn POST | |  | 0.976 |  | 0.027 |  |
| Comm PRE |  | - |  | Comm POST | |  | 0.980 |  | 0.069 |  |
| Pray PRE |  | - |  | Pray POST | |  | 0.976 |  | 0.031 |  |
| Spirit PRE |  | - |  | Spirit POST | |  | 0.978 |  | 0.045 |  |
|  | | | | | | | | | | |
|  | | | | |  |  |  |  |  |  |
|  | | | | |  |  |  |  |  |  |

***Note:*** OR PRE- overall SpSup Scale result before the educational programme; OR POST - overall SpSup Scale result after its completion; Beliefs PRE - beliefs about spirituality before the educational programme; Beliefs POST - beliefs about spirituality after its completion Spirit PRE - spirituality as a phenomenon before the educational programme; Spirit POST - spirituality as a phenomenon after its completion; Recogn PRE - recognition of spiritual suffering before the educational programme; Recogn POST - recognition of spiritual suffering after its completion; Pray PRE - prayer as a support system before the educational programme; Pray POST - prayer as a support system after its completion; Comm PRE - community as a support system before the educational programme; Comm POST - community as a support system after its completion.

The data obtained (Table 2) indicate a deviation from the normal distribution [24]. Therefore, per scientific methodology, the study used the Wilcoxon non-parametric test. The purpose of the Wilcoxon rank test for dependent groups is to determine the magnitude of changes in a given measurement among the subjects and the level of statistical significance of the differences between measurements. Table 3 presents descriptive statistics for two measurements (before the programme – 1 and after its completion – 2) for the overall SpSup Scale result and for individual spiritual competence factors: beliefs regarding spirituality, spirituality as a phenomenon, sensitivity and recognition of suffering, community as a support system, and prayer as a support system. Table 4 presents the results of the Wilcoxon rank test.

| Table 3. Descriptive Statistics: Mean Scores, Standard Deviation, and Median Absolute Deviation | | | | | | | | | | | | | | | | | | | | | |  |  |  |  |  |
| --- | --- | --- | --- | --- | --- | --- | --- | --- | --- | --- | --- | --- | --- | --- | --- | --- | --- | --- | --- | --- | --- | --- | --- | --- | --- | --- |
|  | | **OR1** | | **OR2** | | **Beliefs1** | | **Beliefs2** | | **Spirit1** | | **Spirit2** | | **Recogn1** | | **Recogn2** | | **Pray1** | | **Pray2** | | | **Comm1** | | **Comm2** |  |
| M |  | 63.033 |  | 64.607 |  | 23.656 |  | 24.656 |  | 17.205 |  | 17.590 |  | 5.705 |  | 6.197 |  | 8.041 |  | 7.066 |  | | 8.426 |  | 9.098 |  |
| SD |  | 11.757 |  | 11.394 |  | 4.262 |  | 4.193 |  | 3.593 |  | 3.876 |  | 1.733 |  | 1.364 |  | 4.344 |  | 4.292 |  | | 2.641 |  | 2.348 |  |
| MAD |  | 8.000 |  | 7.500 |  | 3.000 |  | 2.000 |  | 3.000 |  | 2.500 |  | 1.000 |  | 1.000 |  | 3.000 |  | 3.000 |  | | 2.000 |  | 2.000 |  |
|  | | | | | | | | | | | | | | | | | | | | | | | | | |  |

***Note:*** OR1 - overall SpSup Scale result before the educational programme; OR2 - overall SpSup Scale result after its completion; Beliefs1 - beliefs about spirituality before the educational programme; Beliefs2 - beliefs about spirituality after its completion Spirit1 - spirituality as a phenomenon before the educational programme; Spirit2 - spirituality as a phenomenon after its completion; Recogn1 - recognition of spiritual suffering before the educational programme; Recogn2 - recognition of spiritual suffering after its completion; Pray1 - prayer as a support system before the educational programme; Pray2 - prayer as a support system after its completion; Comm1 - community as a support system before the educational programme; Comm2 - community as a support system after its completion; M – mean scores; SD – standard deviation; MAD – median absolute deviation.

Table 4. Comparison of Spiritual Care Competence Levels Expressed in SpSup Scale Results Before and After the Implementation of the ‘Spirituality in Medicine’ Training Module – Wilcoxon Test

|  | | | | | | | | | | | | | | | | | | |  |
| --- | --- | --- | --- | --- | --- | --- | --- | --- | --- | --- | --- | --- | --- | --- | --- | --- | --- | --- | --- |
| **Measur. 1** | |  | | **Measur. 2** | | **W** | | **z** | | **df** | | ***p*** | | **Hodges-Lehmann Estimation** | | **Rank-Two-Series Correlation** | | **Standard Error**  **Rank-two-series correlation** |  |
| OR1 |  | - |  | OR2 |  | 3304.000 |  | -1.000 |  |  |  | 0.318 |  | -1.500 |  | -0.105 |  | 0.104 |  |
| Beliefs1 |  | - |  | Beliefs2 |  | 2702.500 |  | -2.037 |  |  |  | **0.041*** |  | -1.000 |  | -0.217 |  | 0.106 |  |
| Recogn1 |  | - |  | Recogn2 |  | 1828.500 |  | -2.116 |  |  |  | **0.033*** |  | -0.500 |  | -0.246 |  | 0.116 |  |
| Spirit1 |  | - |  | Spirit2 |  | 3061.000 |  | -1.062 |  |  |  | 0.288 |  | -0.500 |  | -0.113 |  | 0.105 |  |
| Pray1 |  | - |  | Pray2 |  | 4172.000 |  | 1.776 |  |  |  | 0.075 |  | 1.000 |  | 0.188 |  | 0.106 |  |
| Comm1 |  | - |  | Comm2 |  | 2765.000 |  | -1.591 |  |  |  | 0.110 |  | -0.500 |  | -0.171 |  | 0.107 |  |
|  | | | | | | | | | | | | | | | | | | |  |
| *Note:* Wilcoxon rank test; rB - Rank-Two-Series Correlation. | | | | | | | | | | | | | | | | | | |  |

**Legend:** OR1 - overall SpSup Scale result before the educational programme; OR2 - overall SpSup Scale result after its completion; Beliefs1 - beliefs about spirituality before the educational programme; Beliefs2 - beliefs about spirituality after its completion Spirit1 - spirituality as a phenomenon before the educational programme; Spirit2 - spirituality as a phenomenon after its completion; Recogn1 - recognition of spiritual suffering before the educational programme; Recogn2 - recognition of spiritual suffering after its completion; Pray1 - prayer as a support system before the educational programme; Pray2 - prayer as a support system after its completion; Comm1 - community as a support system before the educational programme; Comm2 - community as a support system after its completion.

The analyses conducted, along with the application of the Wilcoxon rank test, revealed that for each factor, measurement 1 is lower than measurement 2, except for the prayer factor, for which measurement 1 is higher than measurement 2. Detailed analyses of the overall result and the results in individual subscales indicate the following patterns:

1) For the overall result (pre–post-test) – the mean score in the field the spiritual competence scores among the surveyed students shows a small impact on this area (M = 64.607) compared to the results before the programme was introduced (M = 63.033). The Wilcoxon test results indicate (W = 3304, p < 0.318) that this increase is not statistically significant and the rank-biseriate correlation (rB) = -0.105 indicates also that this is a weak effect.

2) For results on the beliefs about spirituality subscale (pre-post-test) – the educational programme on spirituality beliefs impact on the level of spiritual competence scores in this area (M = 24.656) vis-a-vis the results before the programme (M = 23.656) (W = 2702.5, p < 0.041). Results shows the statistically significant, however the rank-biseriate correlation (rB) = -0.217 indicates that this is a weak effect.

3) For results on the spirituality as a phenomenon subscale (pre-post-test) – the mean score in the subscale of spirituality vis-a-vis one’s own suffering and the suffering of others show a slight increase in the average score at the end of the programme in this area among the surveyed students (M = 17.59) compared to the results before the programme (M = 17.205). The Wilcoxon test results indicate (W = 3061, p < 0.288) that this impact is not statistically significant and the rank-biseriate correlation (rB) = -0.113 indicates that this is a weak effect.

4) For results in the spiritual suffering recognition subscale (pre-post-test) – the spiritual education programme in the area of spiritual suffering recognition does not indicate an increase in competence in this area after programme completion (M = 6.197) compared to the result before its implementation (M = 5.70). The Wilcoxon test and p-value (W = 1828.500, p < 0.033) indicate the values obtained to be statistically significant. However, the rank-biseriate correlation (rB) = -0.246 indicates that this is a weak effect.

5) For results on the community as a support system subscale (pre-post-test) – the mean scores in the subscale of community support has a slight increase in the average score at the end of the programme scores among the surveyed students (M = 9.098) compared to the results before the programme (M = 8.426). The Wilcoxon test results indicate (W = 2765, p < 0.110) that this increase is not statistically significant and the rank-biseriate correlation (rB) = -0.171 indicates that this is a weak effect.

6) For results in the prayer as a support system subscale (pre-post-test) – the educational programme on spirituality in the area of prayer support indicates a lower score in this area among the surveyed students (M = 7.066) compared to the results before the programme (M = 8.041). The Wilcoxon test results indicate (W = 4172, p < 0.075) that this difference is not statistically significant and the rank-biseriate correlation (rB) = 0.188 indicates this to be a weak effect.

In summary, the Wilcoxon t-test results indicate a statistically significant values in SpSup Scale scores in the field of the following subscales: beliefs about spirituality and recognition of spiritual suffering. However, the effect values obtained indicate that the relationship between the training conducted and spiritual competence values is weak in both cases. In the prayer as support subscale, the results indicate a decrease in competence after the training compared to the score before it began. The Wilcoxon test shows that the result is statistically significant but the size effect is small. In the remaining measurements, the results of mean scores show an increase in the second measurement, but these differences are not statistically significant.

Since in the first edition of the programme the module lasted four years, and each year a different topic relevant to the SpSup Scale subscales was covered, at the end of each year, students completed the SpSup Scale subscale as per the topic covered:

- after the second year – beliefs about spirituality and spirituality as a phenomenon.

- after the third year – recognising spiritual suffering.

- after the fourth year – community and prayer as spiritual support.

Concerning measurements for each subscale of the SpSup Scale, to estimate the level of spiritual competence before, during, and after the programme, the ANOVA test for non-parametric tests was used – the Friedman ANOVA test [27]. The choice of test was determined by the results of the Shapiro-Wilk test, as well as the Skewness and Kurtosis for each of the subscales, which indicated a deviation from the normal distribution (Table 5).

Table 5. Shapiro-Wilk Test for Estimating the Normal Distribution for Individual Factors of the SpSup Scale

| **Dependent Variable** |  | **Shapiro-Wilk** | ***p* for Shapiro-Wilk t** | **Skewness** | **Kurtosis** |
| --- | --- | --- | --- | --- | --- |
| Beliefs regarding spirit. | before | 0.932 | **<.001***** | -0.816 | 0.174 |
|  | during | 0.960 | **0.001***** | -0.602 | 0.271 |
|  | after | 0.846 | **<.001***** | -1.940 | 6.142 |
| Spirituality as a phenomenon (Spirituality in relation to one’s own suffering and that of others | before | 0.987 | 0.296 | -0.135 | -0.011 |
|  | during | 0.951 | **<.001***** | -0.808 | 0.550 |
|  | after | 0.987 | 0.294 | -0.062 | -0.214 |
| Spiritual suffering recognition – sensitivity to others’ suffering | before | 0.923 | **<.001***** | -0.512 | 0.923 |
|  | during | 0.917 | **<.001***** | -0.178 | 0.472 |
|  | after | 0.924 | **<.001***** | -0.026 | 0.541 |
| Community as a support system | before | 0.924 | **<.001***** | -0.914 | 0.650 |
|  | during | 0.934 | **<.001***** | -0.660 | 0.147 |
|  | after | 0.926 | **<.001***** | -0.631 | -0.107 |
| Prayer as a support system | before | 0.942 | **<.001***** | -0.242 | -1.152 |
|  | during | 0.956 | **<.001***** | 0.266 | -0.933 |
|  | after | 0.940 | **<.001***** | 0.225 | -1.202 |

To determine significant differences in the analysed measurements, Table 6 presents the results based on Friedman’s ANOVA for each factor, expressed in the SpSup Scale subscales.

Table 6. Comparison of Changes in Spiritual Competence Before, During, and After the Introduction of the Module ‘Spirituality in Medicine’ – Friedman’s ANOVA test

|  | **Before Programme** | | **During Programme** | | **After Programme** | |  |  |  |  |
| --- | --- | --- | --- | --- | --- | --- | --- | --- | --- | --- |
| **Dependent Variable** | **Mdn** | **IQR** | **Mdn** | **IQR** | **Mdn** | **IQR** | **χ2** | ***df*** | ***p*** | **W** |
| Beliefs | 24.000 | 6.000 | 24.500 | 5.000 | 25.000 | 3.000 | 2.874 | 2 | 0.238 | 0.012 |
| Spirituality | 17.000 | 6.000 | 19.000 | 5.000 | 18.500 | 4.000 | 7.472 | 2 | **0.024*** | 0.031 |
| Recognition | 6.000 | 1.000 | 6.000 | 1.000 | 6.000 | 1.000 | 8.898 | 2 | **0.012*** | 0.036 |
| Community | 9.000 | 4.000 | 9.000 | 3.000 | 10.000 | 3.750 | 5.391 | 2 | 0.068 | 0.022 |
| Praying | 9.000 | 8.000 | 7.000 | 5.750 | 6.000 | 8.000 | 6.090 | 2 | **0.048*** | 0.025 |

|  |
| --- |

|  |
| --- |

Friedman’s test analysis indicated that the spirituality programme introduced for medical students has a slight impact on changing spiritual competences. The results show differences in the measurements obtained in the three factors examined in the SpSup Scale subscales: spirituality in relation to one’s own suffering and that of others (χ2=7.47; p<0.05), recognition of spiritual suffering (χ2=8.89; p<0.05), and prayer as a support (χ2=6.09, p<0.05). Concerning spirituality as a phenomenon, the average results obtained during the programme (Mdn = 19) and after its completion (Mdn = 18.5) are significantly higher compared to the average results obtained before the introduction of the module (Mdn = 17). The average results for the factor of recognising spiritual suffering at each stage of measurement are the same (Mdn = 6). The average results for the factor of prayer as a support system indicate the highest results before the introduction of the programme (Mdn = 9) compared to the results during its implementation (Mdn = 7). Both these average values are higher than the results obtained after completing the education on spirituality (Mdn = 6). What is important, in each of the factors presented, the strength of the effect tested using Kendall’s W indicates an insignificant relationship strength. For the remaining factors, Friedman’s test results indicate no significant relationship between the training conducted and the results obtained, and no statistically significant differences between measurements in the following factors: beliefs about spirituality (χ2 = 2.87; p= 0.238), community treated as support (χ2=6.09; p=0.06). The mean values for the factor of beliefs about spirituality show an slightly input in competence in this area during training (Mdn=24.5) and after completion of the programme (Mdn=25) compared to the mean result before its commencement (Mdn=24), but this increase is not statistically significant, and the effect between the variables is small.

According to the literature, when significant relationships are obtained in Friedman’s ANOVA test, a post-hoc method for non-parametric tests is used to determine which pairs of measurements differ statistically significantly from each other (27). If no significant relationships are obtained based on this test, this method should not be used. Some authors suggest that in such a situation, the contrast method may be used [27]. Due to the statistically significant results of the analysis of variance test in the presented study, Conover’s post-hoc pairwise comparison test was applied to these results (Table 7). For the remaining factors – beliefs about spirituality and community as a support system – the contrast method was used (Table 8).

Table 7. Pairwise Comparisons Using Conover’s Post Hoc Test for the Factors of Spirituality

| **Dependent Variable** |  | **t** | **df** | **W_i_** | **W_j_** | **p** | **p_bonf_** | **p_holm_** |
| --- | --- | --- | --- | --- | --- | --- | --- | --- |
| Spirituality as a phenomenon | before – during | 2.719 | 242 | 222.000 | 263.500 | **0.007**** | 0.021 | 0.021 |
|  | before - after | 1.605 | 242 | 222.000 | 246.5000 | 0.110 | 0.329 | 0.219 |
|  | during-after | 1.114 | 242 | 263.500 | 246.500 | 0.266 | 0.799 | 0.266 |
| Sensitivity and Recognition of the spiritual suffering | before-during | 2.746 | 242 | 220.000 | 258.500 | **0.006**** | 0.019 | 0.019 |
|  | before-after | 2.389 | 242 | 220.000 | 253.500 | **0.018*** | 0.053 | 0.035 |
|  | during-after | 0.357 | 242 | 258.000 | 253.500 | 0.722 | 1.000 | 0.722 |
| Prayer as a support system | before-during | 2.452 | 242 | 264.000 | 226.500 | **0.015*** | 0.045 | 0.045 |
|  | before-after | 1.471 | 242 | 264.000 | 241.500 | 0.143 | 0.428 | 0.285 |
|  | during-after | 0.981 | 242 | 226.500 | 241.500 | 0.328 | 0.983 | 0.328 |

The Friedman’ ANOVA test enables us to conclude that the introduction of the module ‘Spirituality in Medicine’ in the medical students’ curriculum has a statistically significant, but a small effect size in the field of beliefs about spirituality, recognition of spiritual suffering and prayer as a support system. Pairwise comparisons using Conover’s post hoc test show that some measures of spiritual competence differ significantly between individual measurement results. Conover’s post hoc comparisons revealed the following patterns:

- In the factor of spirituality as a phenomenon, measurements taken before and during the course of education are statistically significant (t=2.746; p=0.007). The results obtained based on the Bonferroni (pbonf =0.021) and Holm (pholm = 0.021) corrections confirm this significance. The remaining estimated values of the established pairs (pre-post; during-post) were not statistically significant.

- In the factor of recognition of suffering, the data obtained allow us to conclude that the measurements presented in the pair of pre-during (t=2.746; p=0.006) and pre-post (t=2.389; p=0.018) are statistically significant compared to the results determined for measurements during and after training, whose p-value is not statistically significant;

- The results of the Conover test for the prayer as a support system factor indicate a statistically significant relationship for the measurements established in the pre-during education relationship (t=2.452; p=0.015), while the other pairs of measurements are statistically insignificant.

Table 8. Contrast Method for the Factor of Belief in Spirituality and Community as a Method of Support

| **Dependent Variable** | **Comparison** | **Value** | **SD** | **df** | **t** | **p** |
| --- | --- | --- | --- | --- | --- | --- |
| Beliefs about spirituality | before-during | -0.795 | 0.506 | 242 | -1.570 | 0.118 |
|  | during-after | -0.205 | 0.506 | 242 | -0.405 | 0.686 |
| Community as a support system | before-during | -0.631 | 0.309 | 242 | -2.042 | **0.042*** |
|  | during-after | -0.041 | 0.309 | 242 | -0.133 | 0.894 |

In this analysis using the contrast test the results showed that the statistically significant difference occurred in the factor of community treated as a support system in the pair of measurements before and during education (p<0.05). The other measurements, related to beliefs about spirituality and community, as support in the during-after measurement, proved to be statistically insignificant. The result obtained differs from that obtained using Friedman’s ANOVA test and the Wilcoxon test, where the results for this scale were not statistically significant. The difference in the data obtained can be explained by the contrast test used, which some researchers suggest should be employed in cases where Friedman’s ANOVA test yields results that are not statistically significant (27).

**Discussion**

The study aimed principally to determine in what extend whether the introduction of the ‘Spirituality in Medicine’ programme in the general education curriculum for medical students resulted in an increase or decrease in the level of competence in spiritual care among the student participants. The main research question was: to what extent did participation in the ‘Spirituality in Medicine’ programme influence the level of competence in spiritual care, as measured on the SuppScale, among the students taking part in the programme?

The pre- and post-test study (using the Wilcoxon test) showed an impact on results in SpSup Scale scores concerning beliefs about spirituality and recognition of spiritual suffering among the students surveyed after completing the entire programme. The results of those subscales were statistically significant, but the effect values obtained show that the effect of the relationship between the training conducted and spiritual competence values is small in both cases. Interesting results were obtained for the prayer as support subscale, where the level of competence was higher before the training than after its completion. In the remaining measurements, an increase in results was observed in the second measurement, but these differences are not statistically significant.

Results of the Friedman ANOVA test compared changes in spiritual competence before, during, and after the introduction of the ‘Spirituality in Medicine’ module. The analysis show impact in the measurements obtained in the three factors examined in the SpSup Scale subscales: spirituality in relation to one’s own suffering and that of others (χ2=7.47; p<0.05), recognition of spiritual suffering (χ2=8.89; p<0.05), and prayer as support (χ2 = 6.09, p<0.05). The measurement values do indeed indicate statistically significant differences in these subscales but size effect is small.

In this study the contrast test was also used (27). Pairwise comparisons using Conover’s post hoc test show that some measures of spiritual competence differ between individual results for pairs: in the factor of spirituality as a phenomenon before and during education, in the factor of recognising suffering before and during and before and after education, and in the attitude to prayer before and during education. Although the results of the contrast analysis showed a statistically significant difference in the factor of community treated as a support system in the pair of measurements before and during education, the size effect was also small.

In summary, it has been demonstrated that the introduction of the ‘Spirituality in Medicine’ programme in the general education curriculum for medical students has slight impact on the level of knowledge, competence, and sensitivity in spiritual care among the student participants in the areas of spirituality vis-a-vis one’s own suffering and that of others, beliefs regarding spirituality, attitude to community, sensitivity, and recognition of spiritual suffering. The opposite effect was observed for the attitude to prayer, for which the level of competence decreased after the training. The results obtained in this study suggest that the programme should be assessed and evaluated in terms of the proposed exercises or in the context of the students’ independent work on the proposed topics.

In this case, also the influence of the dynamic secularisation process mainly affects the young generation of Polish society [28]. Measurements by the Pew Research Centre show that secularisation processes in Poland are occurring at the fastest rate among the other European countries studied [29, 30]. It can be concluded that the potential for daily prayer has clearly decreased among young people, and is significantly lower than among the adult population (about 40%) [31].

In countries where education in spiritual care has been an integral part of medical curricula, evaluations have been conducted to assess how such programmes influence students’ knowledge and attitudes towards spiritual suffering and spiritual care for their patients [32].

Following the education, students expressed an increased willingness to include religious/spiritual competency in their future practice (p=0.001), were more comfortable sharing their own religious/spiritual beliefs with a patient when appropriate (p=0.02), and were more willing to approach a patient with religious/spiritual concerns (p=0.04) [33]. Osório et al. conducted a randomised controlled trial to evaluate the impact of an educational intervention in ‘spirituality and health’ on students’ knowledge, attitudes, and skills [34]. Students in the intervention group received higher scores on knowledge tests, felt more comfortable and prepared to talk about religious/spiritual beliefs with patients, more readily recognised the importance of hospital chaplains, and more frequently held the opinion that addressing spirituality is important. Students also demonstrated a greater ability to obtain a patient’s spiritual history compared to the control group [34]. Piscitello et al. showed that after the curriculum, residents self-reported increased knowledge regarding the role of chaplains and the training chaplains receive [35]. Bell et al. reported greater awareness of the benefit of clinicians engaging in care for the ‘whole person’ rather than ‘the disease’ [36]. Contributions of other professions to the healing process were acknowledged, and students felt better equipped to discuss spiritual issues with patients. Overall, there is evidence that including spiritual care in the medical education curriculum reduces barriers to spirituality in medicine, enabling skills in history-taking and exploring whole-person care, and participants are also better equipped to understand their own needs for spiritual self-care [32].

In some countries, particularly in the United States and South America, as well as to a lesser extent in the United Kingdom, significant progress has been made in developing and demonstrating the value of spiritual care as part of medical school curricula [32]. For instance, an increase in the spiritual health content in Brazilian medical schools was observed (from 40% in 2011 to 65.5% in 2021). Most medical school representatives agreed that this issue is important in medical training and that more space in the curriculum is needed [37]. However, spirituality in medicine and spiritual care is relatively neglected by the Polish educational system.

**Limitations of the Study**

One of the main limitations of the study based on the pre–post single-group intervention design is the lack of a control group. While the results indicate slight impact on the spiritual competencies among the medical student, the lack of a control group prevents firm conclusions about causality. Factors such as the transition to adulthood, the retest effect or environmental influences may have contributed to the results obtained. A study involving a control group should be conducted, which might reveal the impact of other factors on achieved results. For example in relation to changes in attitudes towards prayer we cannot exclude the fundamental role of social changes in Polish youth. A limitation of the study is the drop-out rate among students during the various phases of the research. As participation in the study was voluntary and anonymous, some of the students who took part in the initial survey did not complete the questionnaire either during the programme or after its conclusion. Another limitation is the concentration in our education on spiritual care, which is currently only for medical students and not for other future healthcare professionals. Potential limitations might also be related to the use of only one tool, the SpSup scale. Using the Emotional Intelligence Scale, for instance, would perhaps provide us with more information about the correlation between emotional intelligence and spiritual competencies. Another potential limitation may be due to the very individual belief what spiritual care means for each student even though we included the definition of spirituality according to PASCiM at the very beginning of the questionnaire (38). As we based our research on self-reported measures, we cannot exclude the role of cognitive processing, baseline psychological distress, readiness to engage in reflection, or social desirability bias such as projecting a favourable image of themselves (although sometimes their use might be unconscious), noted in studies on spirituality in medicine (39, 40, 41, 42). The last potential limitation may be the need to provide online training for almost half of the educational period (for some students during their third year and for all students during their fourth year) due to the COVID-19 pandemic.

**Summary**

In the 2018/2019 academic year, Poland’s first ‘Spirituality in Medicine’ curriculum was launched at a medical university [22]. Our current first longitudinal assessment, conducted over four years, has shown the impact of medical students’ education on their spiritual care competencies. Our preliminary observations and research from other countries support the importance of educating medical students on spirituality and spiritual care and analysing the possibility of introducing changes to the curriculum, especially incorporating clinical practice. Due to small efficacy of our programme we need to consider introducing elements of interpersonal training, enabling students to identify their personality traits, emotional intelligence and strategies, whilst also increasing their self-awareness and sensitivity to the spiritual dimension of life.

**Acknowledgment**

The authors express their sincere gratitude to Mr Miłosz Kuźma and Mr Marcin Pawłowski from the Nicolaus Copernicus University IT Centre in Toruń, Poland for their assistance in preparing the research.

**Author contributions**

MFK. and M.K. contributed to the conception of the study. MFK. collected and analyzed the data. MFK. And MK. drafted the manuscript. MFK. and MK. contributed to the revision of the manuscript. All authors commented on previous versions. All authors read and approved the final manuscript.

**Funding**

This work had been no funding.

**Data availability**

The datasets used and/or analysed during the current study are available from the corresponding author on reasonable request.

**Declarations**

**Ethics approval**

The research project was approved by the Bioethics Committee at Nicolaus Copernicus University in Toruń, Collegium Medicum in Bydgoszcz (KB 736/2018) and the Ethics Committee of the Faculty of Philosophy and Social Sciences at Nicolaus Copernicus University in Toruń (No. 1/2020) in accordance with the Declaration of Helsinki. Ethics committees have approved the research without requiring the participants’ consent.

**Consent to Participate declaration**

Students were informed taking part in study was voluntary and anonymous and all collected answers would be used exclusively for scientific purposes. Their decision to complete the questionnaire was equivalent to their consent to participate in the research. The voluntary and anonymous nature of the study gave participants the option to withdraw from the test at any stage without any consequences. To make research more anonymous, we used also PIN technics, which is described above. Notably, the selected technique did not allow the identification of the respondents.

**Animal or human data**

Not applicable.

**Consent for publication**

Not applicable.

**Competing interests**

The authors declare no competing interests.

**Author details**

**1Małgorzata Fopka-Kowalczyk**, Department of Philosophy and Social Science, Nicolaus Copernicus University in Toruń, Poland; e-mail address: [mfopka-kowalczyk@umk.pl](mailto:mfopka-kowalczyk@umk.pl). Correspondent author.

**2Małgorzata Krajnik,** Department of Palliative Care, Collegium Medicum in Bydgoszcz, Nicolaus Copernicus University in Toruń, Poland; e-mail address: [malgorzata.krajnik@cm.umk.pl](mailto:malgorzata.krajnik@cm.umk.pl)

**Authors' information**

**Małgorzata Fopka-Kowalczyk**, Ph.D., **DSc, Associate Professor at the Nicolau Copernicus University in Toruń.** A psychologist, a pedagogue, a psycho-oncologist, a lecturer at Nicolaus Copernicus University in Poland. She is a graduate of European Palliative Care Academy (EUPCA, 2015-2017), in years 2015-2019 she was a member of the Special Pedagogy Team, functioning at Special Pedagogy Committee of Polish Academy of Science. A co-founder of Academy for Fighting Cancer in Torun. For a number of years she organized National Conferences of Hospice Psychologists. She is a co-author of the curriculum for teaching spirituality in medicine, implemented in the medical study program at NCU’s in Poland. Recent interests have focused on social competence, especially issues of compassion and caring as interpersonal skills, including spiritual support.

**Professor Małgorzata Krajnik**, MD, PhD, Head of Palliative Care Department,

Nicolaus Copernicus University of Toruń, Collegium Medicum in Bydgoszcz, Poland. President of Palliative Association for Spiritual Care in Medicine. Involved in research on palliative care (PC), ethical issues at the end of life and spirituality. Some projects: "Better treatment for breathlessness in palliative and end-of-life care" (Horizon2020; Leader of WP1); as a member of European Association for Palliative Care (EAPC) Task Force on PC for People with Heart Failure – development of position statement; as a member of the Working Group of the Polish Society of Internal Medicine on Medical Futility at Internal Medicine Units – development of position statement on avoiding medical futility in patients dying in a hospital. ,

**References**

1. Puchalski CM, Larson DB. Developing Curricula in Spirituality and Medicine. Acad Med. 1998:73(9):910–4. http://doi.org/10.1097/00001888-199809000-00015.
2. Chida Y, Steptoe A, Powell LH. Religiosity/spirituality and mortality. A systematic quantitative review. Psychother Psychosom. 2009;78:81–90. http://doi.org/10.1159/000190791.
3. Janssen-Niemeijer AJ, Visse M, Van Leeuwen R, Leget C, Cusveller BS. The role of spirituality in lifestyle changing among patients with chronic cardiovascular diseases: a literature review of qualitative studies. J Relig Health. 2017) 56:1460–77. http://doi.org/10.1007/s10943-017-0384-2
4. Wiliams JA, Meltzer D, Arora V, Chung G, Curlin FA. Attention to inpatients’ religious and spiritual concerns: predictors and association with patient satisfaction. J Gen Intern Med. 2011;26:1265–71. http://doi.org/10.1007/s11606-011-1781-y.
5. van Nieuw Amerongen-Meeuse J, Braam A, Anbeek C, Twisk J, Schaap-Jonker H. Treatment alliance and needs of care concerning religiousness and spirituality: a follow-up study among psychiatric inpatients. Int J Soc Psychiatry. 2021;207640211023065. http://doi.org/10.1177/00207640211023065
6. Abu H, Ulbricht C, Ding E, Allison J, Salmoirago-Blotcher E, Goldberg R, et al. Association of religiosity and spirituality with quality of life in patients with cardiovascular disease: a systematic review. Qual Life Res. 2018;27:2777–97. http://doi.org/10.1007/s11136-018-1906-4.
7. Naimi E, Eilami O, Babuei A, Rezaei K, Moslemirad M. The effect of religious intervention using prayer for quality of life and psychological status of patients with permanent pacemaker. J Relig Health. 2020;59:920–7. http://doi.org/10.1007/s10943-018-0698-8.
8. Kazeminezhad B, Tarjoman A, BorjiM. Relationship between praying and selfcare in elderly with heart failure: a cross-sectional study in West of Iran. J Relig Health. 2020;59:19–28. http://doi.org/10.1007/s10943-018-00757-8
9. Abdi A, Soufinia A, BorjiM, Tarjoman A. The effect of religion intervention on life satisfaction and depression in elderly with heart failure. J Relig Health. 2019;58:823–32. http://doi.org/10.1007/s10943-018-0727-7
10. Sobanski P, Krajnik M, Goodlin S. Palliative care for people living with heart disease-does sex make a difference? Front Cardiovasc Med. 2021;8:629752. <http://doi.org/10.3389/fcvm.2021.629752>.
11. Bekelman DB, Dy SM, Becker DM, Wittstein IS, Hendricks DE, Yamashita TE, et al. Spiritual well-being and depression in patients with heart failure. J Gen Intern Med. 2007;22:470–7. <http://doi.org/10.1007/s11606-006-0044-9>.
12. Xing L, Guo X, Bai L, Qian J. Chen J. Are spiritual interventions beneficial to patients with cancer?: a meta-analysis of randomized controlled trials following PRISMA. Medicine (Baltimore). 2018;97:e11948. <http://doi.org/10.1097/MD.0000000000011948>.
13. Durmu¸s M, Ekinci M. The effect of spiritual care on anxiety and depression level in patients receiving hemodialysis treatment: a randomized controlled trial. J Relig Health. 2022;61:2041–55. <http://doi.org/10.1007/s10943-021-01386-4>.
14. Burlacu A, Artene B, Nistor I, Buju S, Jugrin D, Mavrichi I, et al. Religiosity, spirituality and quality of life of dialysis patients: a systematic review. Int Urol Nephrol. 2019;51:839–50. <http://doi.org/10.1007/s11255-019-02129-x>.
15. Tobin ES, Cosiano MF, O’Connor CM, et al. Spirituality in patients with heart failure. JACC Hearth Fail. 2022;10:217–26. http://doi.org/10.1016/j.jchf.2022. 01.014.
16. VanderWeele TJ. On the promotion of human flourishing. *Proc Natl Acad Sci USA*. 2017;114:8148–56. <http://doi.org/10.1073/pnas.1702996114>.
17. Pawlikowski J, Białowolski P. Weziak-Białowolska, D VanderWeele T. Religious service attendance, health behaviors and well-being outcome-wide longitudinal analysis. Eur J Public Health. 2019;29:1177–83. <http://doi.org/10.1093/eurpub/ckz075>.
18. Puchalski CM, Vitillo R, Hull S, Reller N. Improving the spiritual dimension of whole person care: reaching national and international consensus. Palliat Med. 2014;17:642–56. <http://doi.org/10.1089/jpm.2014.9427>.
19. Best M, Leget C, Goodhead A, Paal P. An EAPC white paper on multidisciplinary education for spiritual care in palliative care. BMC Palliat Care. 2020;19:1–11. <http://doi.org/10.1186/s12904-019-0508-4>.
20. Polish Association of Spiritual Care in Medicine, PASCiM. <http://ptodm.org.pl/>. Accessed 23 March 2023.
21. Krajnik M. Whole person care: A hope for modern medicine? Polish Arch Intern Med. 2017:127(10):712–423. <http://doi.org/10.20452/pamw.4100>.
22. Fopka-Kowalczyk M, Groves R, Larkin P and Krajnik M. A training programme for medical students in providing spiritual care to people with advanced diseases and their loved ones: A case study from the Collegium Medicum in Bydgoszcz, Nicolaus Copernicus University in Toruń, Poland. Front. Cardiovasc. Med. 2022;9:909959. <http://doi.org/10.3389/fcvm.2022.909959>.
23. Fopka-Kowalczyk M, Best M, Krajnik M. The Spiritual Supporter Scale as a new tool for assessing spiritual care competencies in professionals: design, validation, and psychometric evaluation. J Relig Health. 2023;62(3):2081-2111. <http://doi.org/10.1007/s10943-022-01608-3>.
24. Ferguson GA, Takane Y. Analiza statystyczna w psychologii i pedagogice.Warszawa: Wyd. PWN; 2003.
25. Guadagno R. Writing up your results – Guidelines based on APA style. PY602, Spring, 2010.
26. Goss-Sampson MA. Statistical analysis in JASP. A Guide for Students. 6th ed. 2024. <https://jasp-stats.org/2024/05/06/statistical-analysis-in-jasp-0-18-3-update-to-the-students-guide-by-mark-goss-sampson/>.
27. Bedyńska S, Brzezicka A. Statystyczny drogowskaz. Praktyczny poradnik analizy danych w naukach społecznych na przykładach z psychologii. Warszawa: Wyd. SWPS; 2007.
28. Kiełb D, Pierzchała M, Gazda M. Youth attitudes towards religious education in Poland. Religions. 2023;14(1):7; doi:[10.3390/rel14010007](https://doi.org/10.3390/rel14010007).
29. Pew Research Center: Being Christian in Western Europe. [http://assets.pewresearch.org/wp-content/uploads/sites/11/2018/05/14165352/Being-Christian-in-Western-Europe-FOR-WEB1.pdf](https://assets.pewresearch.org/wp-content/uploads/sites/11/2018/05/14165352/Being-Christian-in-Western-Europe-FOR-WEB1.pdf) (2018a). Accessed 12 Dec 2022.
30. Pew Research Center: Eastern and Western Europeans Differ on Importance of Religion, Views of Minorities and Key Social Issues. <https://www.pewresearch.org/religion/2018/10/29/eastern-and-western-europeans-differ-on-importance-of-religion-views-of-minorities-and-key-social-issues/> (2018b). Accessed 30 Nov 2024.
31. Mariański J, Słotwińska H, Mąkosa P, et al. Religiosity and recularisation of Polish youth in the 21st century. Quantitative research analysis. Rocznik Teologii Katolickiej. vol XXII:186-202. 2023. <https://repozytorium.uwb.edu.pl/jspui/bitstream/11320/15803/3/RTK_22_2023_J_Marianski_H_Slotwinska_P_Makosa_M_Buk_Cegielka_Religiosity_and_Secularisation_of_Polish_Youth.pdf>. Accessed 30 Nov 2024].
32. Wenham J, Best M, Kissane DW. Systematic review of medical education on spirituality. Intern Med J. 2021;51(11):1781-1790. http://doi.org/10.1111/imj.15421.
33. Smothers ZPW, Tu JY, Grochowski C, Koenig HG. Efficacy of an educational intervention on students’ attitudes regarding spirituality in healthcare: a cohort study in the USA. BMJ Open. 2019;9(4):e026358. <http://doi.org/10.1136/bmjopen-2018-026358>.
34. Osório IHS, Gonçalves LM, Pozzobon PM, Gaspar Júnior JJ, Miranda FM, Lucchetti ALG, Lucchetti G. Effect of an educational intervention in ‘spirituality and health’ on knowledge, attitudes, and skills of students in health-related areas: A controlled randomized trial. Med Teach. 2017;39(10):1057-1064. http://doi.org/10.1080/0142159X.2017.1337878.
35. Piscitello GM, Martin S. Spirituality, religion, and medicine education for internal medicine residents. Am J Hosp Palliat Care. 2020; 37(4):272-277. <http://doi.org/10.1177/1049909119872752>.
36. Bell D, Harbinson M, Toman G, Crawford V, Cunningham H. Wholeness of healing: an innovative Student-Selected Component introducing United Kingdom medical students to the spiritual dimension in healthcare. South Med J. 2010;103(12):1204-9. http://org.doi/ 10.1097/SMJ.0b013e3181f968ce.
37. Lucchetti G, de Araujo Almeida PO, Martin EZ, Góes LG, Cotta KCG, Lima AC, da Silva Ezequiel O, Lucchetti ALG. The current status of ‘spirituality and health’ teaching in Brazilian medical schools: a nationwide survey. BMC Med Educ. 2023;23(1):172. <http://doi.org/10.1186/s12909>.
38. Mascio R, Lynch S, Phillips J, Best M. Predictors of nurses' spiritual care competence: A replication study with Australian palliative care nurses. Palliat Support Care. 2026;28(24):e121. http://doi.org/10.1017/S1478951526102119. PMID: 42047385.
39. Szilagyi C, Palmer PK, Galchutt P, Langstraat K, Fitchett G. Developing emotional intelligence and counselling self-efficacy in clinical pastoral education in healthcare settings: a multicentre pre-post study. Front Med (Lausanne). 2025;10(12):1578653. <http://doi.org/10.3389/fmed.2025.1578653>.
40. de Oliviera Maraldi, E. Response Bias in Research on Religion, Spirituality and Mental Health: A Critical Review of the Literature and Methodological Recommendations. J Relig Health. 2020;59:772-783. http://doi.org/10.1007/s10943-018-0639-6.
41. Pascoal-Carvalho M, Reis-Pina P. Life Review Interventions in Palliative Care: A Systematic Review of Patient-Centered Outcomes. Am J Hosp Palliat Care. 2026:2:10499091251414862. http://doi.org/10.1177/10499091251414862.
42. Rodrigues-Fouto F, Reis-Pina P. Meaning in Life in Palliative Cancer Care: Psychosocial and Existential Outcomes-A Systematic Review. J Palliat Care. 2026:28:8258597261426760. http://doi.org/10.1177/08258597261426760.
